# Effects of Heat Stress and Exogenous Salicylic Acid on Secondary Metabolites Biosynthesis in *Pleurotus ostreatus* (Jacq.) P. Kumm

**DOI:** 10.3390/life12060915

**Published:** 2022-06-17

**Authors:** Yanru Hu, Qianqian Chai, Yue Wang, Yujie Chen, Haozhe Dong, Jinwen Shen, Yuancheng Qi, Haiyou Yu, Fengqin Wang, Qing Wen

**Affiliations:** 1 Key Laboratory of Agricultural Microbial Enzyme Engineering, Ministry of Agriculture, Rural Department, College of Life Sciences, Henan Agricultural University, Zhengzhou 450002, China; huyanru12@163.com (Y.H.); chaixixi@stu.henau.edu.cn (Q.C.); wangyue@stu.henau.edu.cn (Y.W.); yujiec@stu.henau.edu.cn (Y.C.); donghaozhe@stu.henau.edu.cn (H.D.); shenjinwen369@163.com (J.S.); qiyuancheng@henau.edu.cn (Y.Q.); w_fengqin@henau.edu.cn (F.W.); 2 College of Food and Bioengineering, Henan University of Animal Husbandry and Economy, Zhengzhou 450002, China; yuhaiyou1985@163.com

**Keywords:** environmental factors, secondary metabolites, *Pleurotus ostreatus*, metabolomics, transcriptomics

## Abstract

*Pleurotus ostreatus* (Jacq.) P. Kumm has high medicinal value, but few studies exist on regulating secondary metabolite biosynthesis. Environmental factors play a substantial role in the accumulation of microbial secondary metabolites. In this study, the effects of heat stress (24 h) and salicylic acid (0.05 mmol/L) treatment on the secondary metabolism of *P. ostreatus* were analyzed by metabolome, transcriptome, and gene differential expression analysis. Metabolome and transcriptome analyses showed that salicylic acid significantly increased the accumulation of antibiotics and polyketones, while heat stress increased the accumulation of flavonoids, polyketones, terpenoids, and polysaccharides. The content and the biosynthetic genes expression of heparin were markedly increased by heat stress, and the former was increased by 4565.54-fold. This study provides a reference for future studies on secondary metabolite accumulation in edible fungi.

## 1. Introduction

The medicinal properties of fungi have been incorporated into health practices for a considerable portion of history. Many fungal secondary metabolites (such as penicillin, statins, and polysaccharide) can contribute to extending human life [1]. Fungi are remarkable microbial cell factories for secondary metabolite production, such as their strong and abundant secondary metabolite production capacity and chemical diversity [2]. Mushrooms produce various medicinal compounds, which play important roles in their medicinal properties, including antibacterial, anti-inflammatory, anticholesterolemic, radical scavenging, etc. [3]. Mushrooms are also rich in fiber, protein, vitamins, mineral elements, etc. Consequently, the mushroom has become a functional food, and its abundant secondary metabolites have become a new source of natural active substances [4].

Secondary metabolites are considered important resources for novel pharmaceuticals development, but there are still unsolved problems. One major problem is discovering new medicinal active substances to address antibiotic resistance [5]. Another significant issue is the low production of many important metabolites, which limits their widespread application [6]. Environmental factors affect secondary metabolism. Therefore, many studies have been performed to improve secondary metabolism biosynthesis by changing the culture conditions. Physical means (temperature, solid culture and pH change, etc.) have been reported to rearrange metabolites and increase the accumulation of secondary metabolites [7,8]. Single chemical/biological or biochemical signals, such as salicylic acid (SA) and ethylene, can also regulate secondary metabolism [9,10]. Additionally, nutritional conditions, including carbon sources (e.g., microcrystalline cellulose, D-galactose), nitrogen sources (e.g., ammonium sulfate, glycine, glutamine, and asparagine), and the ratio of carbon and nitrogen can also regulate secondary metabolism [11,12,13].

*Pleurotus ostreatus* (Jacq.) P. Kumm is one of the most commercially cultivated edible fungi worldwide [14]. As a wood-rotting fungus, *P. ostreatus* can degrade various lignocellulosic substrates that are largely produced as by-products in agricultural, forest, and food-processing industries. *P. ostreatus* has high nutritional value and is an excellent source of crude fiber, beta-glucan, and amino acids [15]. In addition, *P. ostreatus* also has important medicinal values as it contains many bioactive components, such as polysaccharide, steroids, flavonoids, coumarin metabolites, scalarane sesterterpenes, unusual sesterterpenes, and naphtho-γ-pyrone compounds [6,15,16,17,18]. However, there are few studies on the regulation of the accumulation of secondary metabolites in *P. ostreatus*.

Heat shock (HS), one of the most important physical environmental factors that impact the development of *P. ostreatus* [19], has been used to improve ganoderic acid biosynthesis in *Ganoderma lucidum* (Curtis) P. Karst [7]. In a previous study, we discovered that an appropriate amount of exogenous SA (0.05 mmol/L) significantly improved the HS resistance of *P. ostreatus* [20]. However, the effects of HS and exogenous SA on the biosynthesis of the secondary metabolites in *P. ostreatus* are still unclear. In this study, the difference in secondary metabolite contents between treatments with and without heat stress (HS/no HS), and between treatments with and without the addition of exogenous SA (no HS SA/no HS) were compared through data-mining of the metabolome and transcriptome of *P. ostreatus* [20] and gene differential expression analysis. This study can lay a theoretical foundation for developing *P. ostreatus* rich in secondary metabolites and provide a reference for identifying new secondary metabolites from edible fungi.

## 2. Materials and Methods

### 2.1. Strains and Culture Medium

The *Pleurotus ostreatus* New 831 strain used in this study was obtained from Applied Fungi Laboratory, College of Life Science, Henan Agricultural University. The mushroom complete medium (glucose 20 g/L, VB1 0.01 g/L, MgSO_4_·7H_2_O 0.5 g/L, KH_2_PO_4_ 1 g/L, yeast extract 5 g/L, agar 2%) was used to cultivate the mycelium of *P. ostreatus*.

### 2.2. Salicylic Acid Treatment

The mother liquor (1 mol/L) of salicylic acid (Sigma, St. Louis, MO, USA) was prepared in ethanol and sterilized by filtration. This salicylic acid solution was diluted into the mushroom complete medium to obtain a final concentration of 0.05 mmol/L [20]. Water was used as control; then, an activated mycelial block (5 mm) was inoculated in the mushroom complete medium with and without SA for 5 d at 25 °C. Six biological replicates for each treatment were collected for metabolome analysis and three biological replicates for each treatment were collected for transcriptome analysis.

### 2.3. Heat Stress Treatment

For 5 days at 25 °C, an activated mycelial block (5 mm) was inoculated in the mushroom complete medium. The *P. ostreatus* mycelium was then subjected to heat stress (40 °C for 24 h). No-heat-stress treatment was used as a control. Finally, the samples were collected and stored at −70 °C until use [19]. Six biological replicates for each treatment were used for metabolome analysis and three biological replicates for each treatment were used for transcriptome analysis.

### 2.4. Metabolome Analysis

The samples were extracted and analyzed with LC-TOF-MS according to a previous report [20]. Briefly, the mycelium was extracted with methanol:acetonitrile (2:1, *v*/*v*), then the samples were placed in ice water for ultrasonic lysis for 10 min. After depositing at −20 °C for 2 h, the lysates were centrifuged at 4 °C for 30 min (4000 rpm). After adding 300 µL of supernatant into 96-well plate, the plate was dried in a freeze dryer. Further, 300 µL methanol (10%) was added to suspend the sample, and the sample was ultrasound treated for 10 min in ice water. After centrifuging at 30,000× *g* and 4 °C for 20 min, 250 µL supernatant was taken for LC/TOF–MS analysis. Six biological replicates for each treatment were included in this metabolomics study. Metabolomics data were deposited to the EMBL-EBI MetaboLights database with the identifier MTBLS3836. The complete dataset can be accessed here at https://www.ebi.ac.uk/metabolights/MTBLS3836 (accessed on 1 June 2022).

### 2.5. Transcriptome Analysis

The samples were extracted and analyzed with MGISEQ-based RNA sequencing according to a previous report [20]. Briefly, total RNA was isolated from mycelium and then was used to synthesize the DNA libraries. MGISEQ-based RNA sequencing was performed commercially on the MGISEQ-2000 RS platform. The reads of low quality, joint contamination, and high content of unknown base N were removed to obtain clean reads. The *P. ostreatus* genome (PC15 v2.0) was used to map with the clean reads using HISAT and Bowtie2. Then, using RSEM, the expression levels of genes were calculated, and the expression difference level was examined using DEGseq. Thresholds of fold change ≥ 2 and adjusted *p* ≤ 0.001 were applied to assess the significant differences in transcript levels. The sequencing data can be obtained with the accession number SUB10696145 in the NCBI Sequence Read Archive (SRA) database.

### 2.6. Gene Expression Analysis

To access the expression levels of genes, total RNA was extracted using the RNAiso™ Plus (TaKaRa, Dalian, China) according to the manufacturer’s protocol. The cDNA was synthesized from the total RNA using a PrimeScript RT Reagent Kit with gDNA Eraser (TaKaRa, Dalian, China). Subsequently, the RT-qPCR assay was performed according to a previous report [21]. Actin was used as an internal control [21]. All the primers used in this experiment are listed in Supporting Information Table S2. The relative expression levels of the genes were determined using the 2^−ΔΔCT^ method [22].

### 2.7. Significance Analysis

One-way analysis of variance (ANOVA, GraphPad Software, Inc., San Diego, CA, USA) and Tukey’s test (SPSS, Version 26.0, IBM Corporation, Armonk, NY, USA) were used to analyze the results. Similar results were obtained in three independent experiments. Each result was expressed as the mean ± SD, and *p* < 0.05 was considered statistically significant.

## 3. Results

### 3.1. Metabolome Analysis

**Figure 1:**
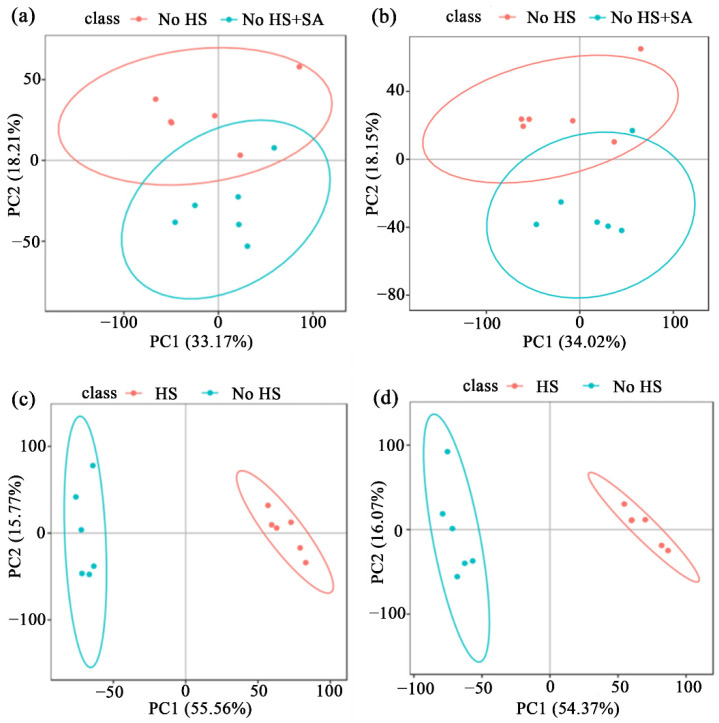
Metabolome analysis. (**a**) Principal component analysis (PCA) was used to separate these metabolites in No HS+SA/No HS group. (**b**) Orthogonal projection to latent structure with discriminant analysis (OPLS-DA) was used to separate these metabolites in No HS+SA/No HS group. (**c**) PCA was used to separate these metabolites in HS/No HS group. (**d**) OPLS-DA was used to separate these metabolites in HS/No HS group.

To determine the effects of HS and SA on the secondary metabolism of *P. ostreatus*, metabolome analysis was performed according to our previous report [20]. Principal component analysis (PCA) and orthogonal projection to latent structure with discriminant analysis (OPLS-DA) were used to distinguish metabolites between different treatments. Results are shown in Figure 1 and Table S3. Different metabolites and their levels can be identified between the two groups. This suggests the reliability of the models and, thus, the data can be used for subsequent analyses.

### 3.2. Metabolic Changes and the Metabolic Pathway Analysis

In total, 132 metabolites changed significantly in the negative mode after SA treatment, while 118 metabolites changed significantly in the positive mode. Heat-shock treatment caused significant changes in 2114 metabolites in the negative mode and 1938 metabolites in the positive mode. There were more kinds of metabolites detected in the negative mode as compared to the positive mode; hence, the metabolites in the negative mode were analyzed subsequently.

Upon SA treatment, 61 metabolites increased significantly (Table S1). The most abundant metabolites included antibiotics (eight metabolites) and polyketides (eight metabolites), followed by benzene and derivatives (seven metabolites), amino acids, peptides and analogs (five metabolites), carbohydrates (four metabolites), organic acids (three metabolites), terpenoids (two metabolites), fatty acyls (two metabolites), glycerophospholipids (two metabolites), nucleic acids and analogs (two metabolites), alkyl fluorides (two metabolite), amines (one metabolite), glycerolipids (one metabolite), phenols and derivatives (one metabolite), purines and derivatives (one metabolite), sphingolipids (one metabolite), and sterol lipids (one metabolite). There were 11 unclassified metabolites, which were increased significantly. The metabolite with the largest fold increase was dichlorphenamide (554.86-fold), followed by selenohomocystine (96.94-fold), 4-hydroxybenzoic acid (45.77-fold), 2-pyrocatechuic acid (27.09-fold), cefozopran (24.05-fold), 2-oxo-3-hydroxy-4-phosphobutanoic acid (19.73-fold), trifluoroacetic acid (18.05-fold), kaempferol 3-glucuronide-7-sulfate (17.30-fold), 5-sulfo-1,3-benzenedicarboxylic acid (14.47-fold), and 3S-bromobutanoic acid (13.12-fold). The fold increase in the remaining metabolites was less than 10.

**Figure 2:**
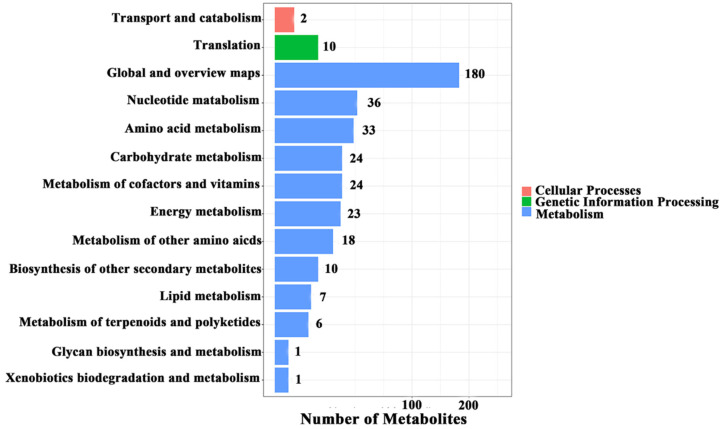
KEGG cluster analysis of metabolites of HS/No HS group.

Upon heat shock treatment, 1092 metabolites increased significantly. KEGG pathway analysis showed that “Metabolism of cofactors and vitamins”, “Biosynthesis of other secondary metabolites”, “Metabolism of terpenoids and polyketides”, and “Glycan biosynthesis and metabolism” were changed significantly (Figure 2). To analyze the effect of heat shock on metabolism, the top 50 metabolites were further analyzed (Table S2). The most abundant metabolites were fatty acyls (nine metabolites), followed by polyketides (seven metabolites), flavonoids (six metabolites), purines and derivatives (four metabolites), carbohydrates (three metabolites), nucleic acids and analogs (three metabolites), amino acids, peptides, and analogs (two metabolites), coumarins and derivatives (one metabolite), prenol lipids (one metabolite), quinone (one metabolite), sphingolipids (one metabolite), and terpenoids (one metabolite). Ten unclassified metabolites were also found. Among these metabolites, heparin (a polysaccharide) showed the largest fold increase (4565.54-fold).

### 3.3. Transcriptome Analysis

**Figure 3:**
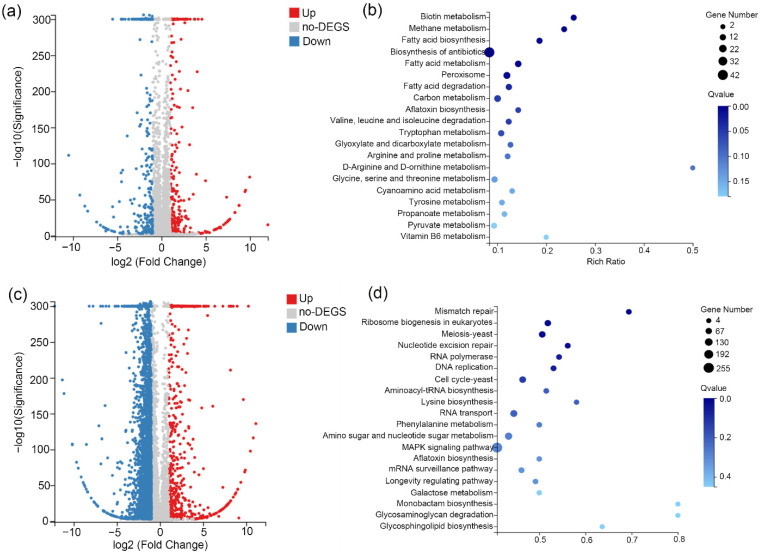
Transcriptome changes and KEGG pathway analysis. (**a**) The volcano figure of No HS + SA/No HS group. (**b**) The top 20 metabolic pathways with significant changes (sorted by Q value) of No HS + SA/No HS group. (**c**) The volcano figure of HS/No HS group. (**d**) The top 20 metabolic pathways with significant changes in HS/No HS group.

To analyze the gene expression changes, transcriptome responses were further analyzed. Upon SA treatment, 265 genes were significantly up-regulated and 265 genes were significantly down-regulated (Figure 3a). The top 20 metabolic pathways with significant changes (Sort by Q value) in KEGG pathway enrichment analysis were found to be “Biotin metabolism”, “Methane metabolism”, “Biosynthesis of antibiotics”, “peroxisome”, and many primary metabolisms, such as fatty acid metabolism, carbon metabolism, amino acid metabolism, and so on (Figure 3b). Upon heat-shock treatment, 639 genes were significantly up-regulated and 3660 genes were significantly down-regulated (Figure 3c). The top 20 metabolic pathways with significant changes in KEGG pathway enrichment analysis were involved in complex physiological processes, such as “Mismatch repair”, “Ribosome biogenesis in eukaryotes”, “MAPK signaling pathway”, and so on (Figure 3d).

### 3.4. Putative Secondary Metabolic Pathway Analysis

**Figure 4:**
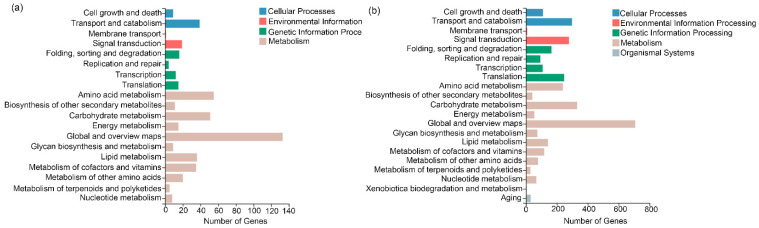
KEGG cluster analysis of differential genes. (**a**) KEGG cluster analysis of differential genes in No HS+SA/No HS group. (**b**) KEGG cluster analysis of differential genes in HS/No HS group.

The results of KEGG pathway classification were analyzed to precisely target secondary metabolic pathways. Upon SA and heat-shock treatment, the secondary metabolic pathways mainly included “Biosynthesis of other secondary metabolites”, “Glycan biosynthesis and metabolism”, “Metabolism of cofactors and vitamins”, and “Metabolism of terpenoids and polyketides” (Figure 4a,b).

**Figure 5:**
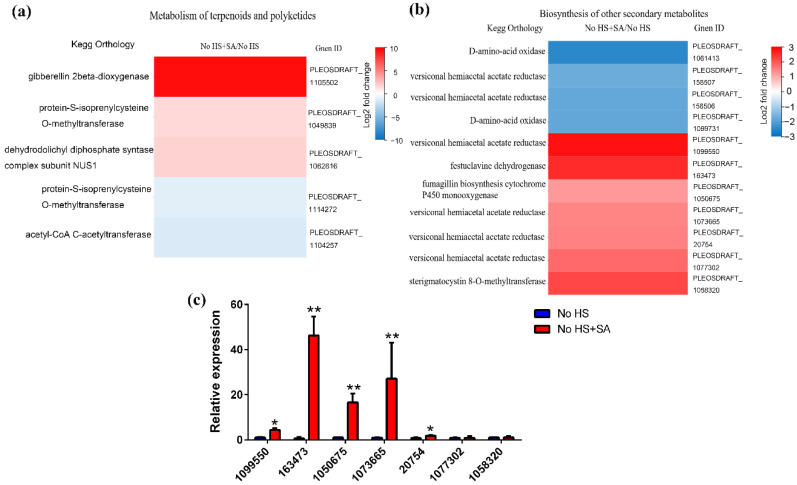
Gene expression analysis of secondary metabolic pathway upon SA treatment. (**a**) Gene expression analysis of “Metabolism of terpenoids and polyketides”. (**b**) Gene expression analysis of “Biosynthesis of other secondary metabolites”. (**c**) qRT-PCR verification of changes in gene expression of “Biosynthesis of other secondary metabolites”. Results are expressed as the means ± SD (*n* = 3). Data are the mean of three independent measurements ± the standard deviation indicated by the error bars, * *p* < 0.05 and ** *p* < 0.01 using the one-way ANOVA test.

Following SA treatment, we concentrated on “Metabolism of Terpenoids and Polyketides” and “Biosynthesis of Other Secondary Metabolites”. Transcription levels of five genes were changed significantly upon SA treatment, among which three genes showed an increase (putative gibberellin 2beta-dioxygenase, protein-S-isoprenylcysteine O-methyltransferase, and dehydrodolichyl diphosphate synthase) and two genes in “Metabolism of terpenoids and polyketides” showed a decrease (Figure 5a). The expression levels of 11 genes in “Biosynthesis of other secondary metabolites” contributing to antibiotic synthesis were significantly changed. Seven genes showed a significant increase in transcription levels, including putative versiconal hemiacetal acetate reductase (four numbers), putative festuclavine dehydrogenase, putative fumagillin biosynthesis cytochrome P450 monooxygenase, and putative sterigmatocystin 8-O-methyltransferase (Figure 5b). qRT-PCR results were consistent with the transcriptome analysis results (Figure 5c).

**Figure 6:**
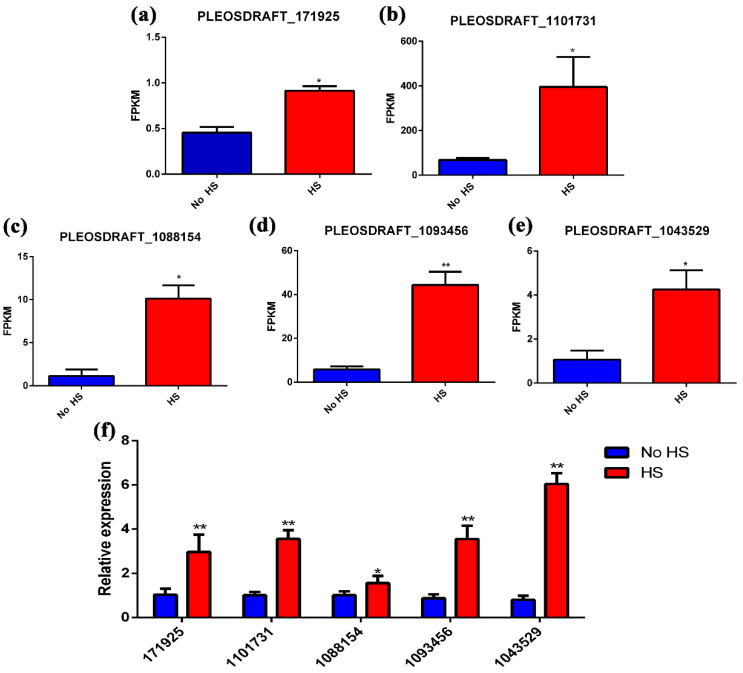
Gene expression analysis of “Glycan biosynthesis and metabolism” upon heat-shock treatment. (**a**–**e**) Gene expression analysis of PLEOSDRAFT_171925 (putative cysteine desulfurase), PLEOSDRAFT_1101731 (putative oligosaccharyltransferase complex subunit alpha), PLEOSDRAFT_1088154 (putative oligosaccharyltransferase complex subunit delta), PLEOSDRAFT_1093456 (putative dolichyl-phosphate-mannose-protein mannosyltransferase), and PLEOSDRAFT_1043529 (putative α-galactosidase). (**f**) qRT-PCR verification of the expression changes in these genes. Results are expressed as the means ±SD (*n* = 3). Data are the mean of three independent measurements ±the standard deviation indicated by the error bars, * *p* < 0.05 and ** *p* < 0.01 using the one-way ANOVA test.

We focused on “Glycan biosynthesis and metabolism” after heat-shock treatment to analyze heparin biosynthesis. Results showed that the transcription levels of five genes were significantly increased, including PLEOSDRAFT_171925 (putative cysteine desulfurase), PLEOSDRAFT_1101731 (putative oligosaccharyltransferase complex subunit alpha), PLEOSDRAFT_1088154 (putative oligosaccharyltransferase complex subunit delta), PLEOSDRAFT_1093456 (putative dolichyl-phosphate-mannose-protein mannosyltransferase), and PLEOSDRAFT_1043529 (putative α-galactosidase) (Figure 6a–e). qRT-PCR results were consistent with the transcriptome analysis results (Figure 6f).

## 4. Discussion

Since ancient times, fungi have played an important role in healthcare products. Many fungal secondary metabolites (penicillin, statins, and so on) have been used to treat diseases and extend people’s lives [1]. Fungi are a rich source of secondary metabolites because of their strong and abundant secondary metabolite production capacity [2]. However, the lack of basic research and knowledge on gene function is hindering the exploitation of these metabolites [2]. Furthermore, extracting small amounts of naturally accumulated secondary metabolites is difficult. As a result, appropriate methods for increasing the content of secondary metabolites in fungi must be developed.

Scientists acquire genome data with powerful tools to study the molecular genetics of interactions between the secondary metabolism of fungus and the environment [23]. Environmental factors are known to affect secondary metabolism. Many studies have focused on enhancing secondary metabolite biosynthesis by changing the culture conditions through physical approaches (heat stress, solid culture, pH change, etc.), by treatment with single chemical/biological or biochemical signals (MeJA, SA, phenobarbital, etc.), and by changing nutritional conditions (microcrystalline cellulose, D-galactose, etc.) [7,8,9,11,24,25]. Temperature is one of the main environmental factors that may unexpectedly disrupt macrofungi growth and development. Heat stress affects cell metabolism, especially primary metabolism, such as tricarboxylic acid, amino acid metabolism, glycolysis, etc. [20]. Appropriate heat stress is beneficial to macrofungi in terms of nutrient accumulation, and such stress can be used to improve the accumulation of specific metabolites in cells. [26]. As secondary metabolism comes from primary metabolism, heat stress also promotes the accumulation of valuable secondary metabolites, such as ganoderic acid and polysaccharides [26]. *P. ostreatus* is wildly cultivated in the world and contains many bioactive substances. However, there are only a few studies on the regulation of secondary metabolite accumulation in *P. ostreatus*. In our previous study, heat stress and exogenous salicylic acid altered the primary metabolism of *P. ostreatus* [20]. In the present study, the effects of heat stress (24 h) and salicylic acid (SA) (0.05 mmol/L) treatment on the secondary metabolism of *P. ostreatus* were analyzed by data-mining of the metabolome and transcriptome uploaded in our previous study. The results showed that the exogenous addition of SA could significantly promote the accumulation of antibiotics and polyketones. At the same time, heat shock could significantly increase the content of flavonoids, polyketones, terpenoids, and polysaccharides (especially heparin).

The rise of antibiotic resistance poses a serious threat to modern health care. It is vital to develop new drugs to treat drug-resistant infections. Soil-dwelling actinomycetes are the major source of antimicrobial compounds, which are currently being used in the clinic [27]. Fungi also produce antibiotics during their defense process [28]. In the present study, SA treatment significantly increased the levels of cefozopran (24.05-fold), cephamycin C (9.46-fold), tetracenomycin (6.83-fold), fortimicin (5.38-fold), dihydroalbocycline (4.30-fold), 2-heptyl-4-quinolone (2.65-fold), protomycinolide IV (2.41-fold), and rimocidine (2.35-fold). Cefozopran and cephamycin C are β-lactam antibiotics that inhibit bacterial cell-wall synthesis [28]. Tetracenomycin and dihydroalbocycline are polyketide antibiotics [29,30]. Fortimicin is an aminoglycoside antibiotic [31]. 2-Heptyl-4-quinolone is a kind of tetrabromopyrrole, which has been shown to exhibit antibiotic activity against human and marine bacteria [32]. Protomycinolide IV and rimocidine are macrolide antibiotics [33]. Transcriptome analysis showed that SA increased the expression levels of seven genes involved in antibiotic synthesis. Four out of seven genes were versiconal hemiacetal acetate reductases, which convert versiconal hemiacetal acetate to versiconal, and versiconol acetate to versiconol in aflatoxin biosynthesis [34]. Sterigmatocystin 8-O-methyltransferase has also been reported to be involved in aflatoxin biosynthesis [35]. However, these genes may be involved in the synthesis of antibiotics other than aflatoxins in *P. ostreatus*. Festuclavinedehydrogenase, a P450 monooxygenase, which has been characterized in the *N. fumigata* gene cluster, is required for the conversion of festuclavine to different alkaloids [36]. Cytochrome P450 monooxygenase is the most versatile biocatalyst in nature that plays an important role in many biosynthesis and biodegradation processes, involved in a wide range of secondary metabolic pathways. Thus, cytochrome P450 monooxygenases are potential candidates in the synthesis of different antibiotics [37]. Antibiotics and their synthesis genes have not been reported in *P. ostreatus*. Our result showed that SA might induce the accumulation of antibiotics and transcription of their related genes in *P. ostreatus*.

Heparin is a naturally occurring glycosaminoglycan that is used as an anticoagulant drug in the clinic. Heparin could inhibit the cellular invasion by SARS-CoV-2 [38]. Heparin comes mainly from livestock, principally the porcine intestine. One limitation of heparin production is that it is based on a single animal species, and there are potential issues with heparin derived from animals. The 2008 pollution crisis prompted the search for new animal sources and a deep investigation into non-animal sources of heparin. In the past five years, new animal-derived, chemical, and chemical enzyme methods have been introduced for the preparation of heparin drugs [39]. To our knowledge, there has been no report about fungi-produced heparin. Our results showed that heat shock significantly increased heparin content (4565.54-fold) in the mycelium of *P. ostreatus*. Heparin is biosynthesized by a series of transferases, such as xylosyltransferase, galactosyltransferase, glucuronosyltransferase, N-sulfotransferase, and so on [39]. Transcriptome analysis showed that the expression of PLEOSDRAFT_171925 (a putative cysteine desulfurase), PLEOSDRAFT_1101731 (a putative oligosaccharyltransferase complex subunit alpha), PLEOSDRAFT_1088154 (a putative oligosaccharyltransferase complex subunit delta), PLEOSDRAFT_1093456 (a putative dolichyl-phosphate-mannose-protein mannosyltransferase), and PLEOSDRAFT_1043529 (a putative α-galactosidase) in “Glycan biosynthesis and metabolism” have increased significantly. Three genes belonging to glycosyltransferase suggested that these genes may participate in the formation of heparin. Cysteine is a sulfur source for the biosynthesis of many sulfur-containing biomolecules. Cysteine desulfurase plays an important role in this process and participates in the trafficking of sulfur for various metabolic pathways [40]. In addition, sulfur-containing amino acids could also act as sources for sulfation [41]. High levels of sulfation are frequently found in heparin [40]. It suggested that heat shock induced cysteine desulfurase transcription to provide sulfur for the sulfation of heparin. α-Galactosidase catalyzes the hydrolysis of α-galactose residues present at the terminal of galactose oligosaccharides. Under suitable conditions, α-galactosidases can also catalyze transglycosylation reactions [42]. The present study revealed the existence of a possible heparin synthesis pathway in fungi for the first time, which provided a non-animal source of heparin.

## 5. Conclusions

Culture conditions, signaling molecules, the nutritional composition of the medium, and other factors all can affect the secondary metabolism of microorganisms. However, different factors have different effects on the biosynthesis of secondary metabolites. The effects of HS and exogenous SA addition on secondary metabolite biosynthesis in *P. ostreatus* were investigated using metabolome, transcriptome, and gene differential expression analysis. The result showed differences in the accumulation of secondary metabolite between HS and exogenous SA. HS treatment can significantly increase the accumulation of flavonoids, polyketones, terpenoids, and polysaccharides, among which the content of the anticoagulant drug heparin was increased by 4565.54-fold. Being different from HS treatment, the exogenous addition of SA leads to the remarkable accumulation of antibiotics and polyketones. Therefore, appropriate treatment conditions can be adopted to promote the biosynthesis of some special secondary metabolites in *P. ostreatus*. Furthermore, the biosynthetic pathways of most secondary metabolites in *P. ostreatus* are not fully understood and are worth further exploring.

## Data Availability

The complete metabolome dataset can be accessed here https://www.ebi.ac.uk/metabolights/MTBLS3836 (accessed on 1 June 2022). The sequencing data were deposited in the NCBI Sequence Read Archive (SRA) database under the accession number SUB10696145.

## Supplementary Materials

The following supporting information can be downloaded at: https://www.mdpi.com/article/10.3390/life12060915/s1, Table S1: Metabolites with significantly increased content upon salicylic acid treatment; Table S2: Metabolites with significantly increased content (top 50) upon heat-shock treatment; Table S3: OPLS-DA results of LC-MS data in different groups. R2 > 0.5 and Q2 > 0.5 indicated a good model; Table S4: qRT-PCR primers used in this study.
