# Supplementary material for: Effects of Heat Stress and Exogenous Salicylic Acid on Secondary Metabolites Biosynthesis in Pleurotus ostreatus (Jacq.) P. Kumm
Source: Life (Basel). 2022 Jun 17;12(6):915. doi: 10.3390/life12060915 (PMC9225297; doi:10.3390/life12060915)
Supplement: Supplementary file 1 [file life-12-00915-s001.zip › life-1770779-supplementary.pdf]

Supplementary Material for Life

Effects of heat stress and exogenous salicylic acid on secondary metabolites biosynthesis in *Pleurotus ostreatus* (Jacq.) P. Kumm

Yanru Hu[1], Qianqian Chai[1], Yue Wang[1], Yujie Chen[1], Haozhe Dong[1], Jinwen Shen[1], Yuancheng Qi[1], Haiyou Yu[2], Fengqin Wang[1], Qing Wen[1]*

Affiliations: 1: Key Laboratory of Agricultural Microbial Enzyme Engineering, Ministry of Agriculture, Rural Department, College of Life Sciences, Henan Agricultural University, Henan, Zhengzhou 450002, People's Republic of China.

2: College of Food and Bioengineering, Henan University of Animal Husbandry and Economy, Henan, Zhengzhou 450002, People's Republic of China.

**Corresponding author. Mailing address: College of Life Sciences, Henan Agricultural University, 95 Wenhua Road, Zhengzhou 450002, People's Republic of China. Telephone and Fax: 0371-63555790. E-mail: wenqing@henau.edu.cn*

Supplementary Material

Table S1. Metabolites with significantly increased content under salicylic acid treatment.

Family	Number	Description	ratio
Alkyl fluorides	1	Perfluorononanoic acid	7.19
Amines	1	N-Lactoyl ethanolamine	2.02
Amino acids, peptides, and analogues	5	Selenohomocystine	96.94
		Stepronin	9.33
		Selenocystine	3.55
	8	Gamma-glutamyl-Gamma-glutamate	3.46
Antibiotics		Neuromedin B	2.24
		Cefozopran	24.05
		Cephamycin C	9.46
		Tetracenomycin	6.83
		Fortimicin	5.38
		Dihydroalbocycline	4.30
		2-Heptyl-4-quinolone	2.65
	7	Protomycinolide IV	2.41
Benzene,and derivatives		Rimocidine	2.35
		Dichlorphenamide	554.86
		4-Hydroxybenzoic acid	45.77
		2-Pyrocatechuic acid	27.09

		5-Sulfo-1,3-benzenedicarboxylic acid	14.47
		Thaumatin b	2.98
Carbohydrates	4	6-Hydroxy-5-[(4-sulfophenyl)azo]-2-naphthalenesulfonic acid	2.86
		Azorubine	2.28
		Palatinose	5.68
		beta-N-Acetylglucosamine	4.49
Fatty Acyls	2	Stachyose	3.76
		D-erythro-D-galacto-octitol	1.81
Glycerolipids	1	3S-bromobutanoic acid	13.12
Glycerophospholipids	2	6,9,12,15,18-Tetracosapentaynoic acid	7.23
		Monoglyceride citrate	1.91
Nucleic acids and	2	PS(20:5/20:4)	3.93
analogues		PC(o-24:0/20:4)	1.92
Organic acids	3	Cytarabine	2.47
		CDP-Ethanolamine	1.60
		2-Oxo-3-hydroxy-4-phosphobutanoic acid	19.73
Phenols and derivatives	1	Trifluoroacetic acid	18.05
Polyketides		Homoanserine	1.88
	8	{[1-(4-hydroxy-3-methoxyphenyl)-3-oxobutan-2-yl]oxy}sulfonic acid	1.57
		Kaempferol 3-glucuronide-7-sulfate	
		Velloquercetin 3,3',4'-trimethyl ether	17.30
		Kaempferol 3,7,4'-tri-O-sulfate	5.42
		{2-hydroxy-5-[3-(4-methoxy-1-benzofuran-5-yl)-3-oxopropanoyl]phenyl	3.00
		}oxidanesulfonic acid	3.55
		Vitexin 3''',4'''-Di-O-acetyl 2''-O-rhamnoside	
		3'-Bromo-6'-hydroxy-2',4,4'-trimethoxychalcone	3.17
Purines and derivatives		Malvidin 3-(6''-acetylglucoside)-5-glucoside	3.09
Sphingolipids	1	Quercetagetin 4'-methyl ether 7-(6-(E)-caffeylglucoside)	1.96
Sterol Lipids		7-Methylguanosine	1.88
Terpenoids	1		3.59
	1	FMC-6(d18:1/22:0(2-OH))	
other	2	Cholestane-3,7,12,25-tetrol-3-glucuronide	1.96
		Umbelliprenin	7.28
	11	Inumakilactone A glycoside	3.54
		6-Oxo-2-hydroxy-7-(4'-chlorophenyl)-3,8,8-trichloroocta-2E,4E,7E-trien	2.12
		oate	7.29
		di-trans,poly-cis-Octaprenyl diphosphate	
		2-C-Methyl-D-erythritol 4-phosphate	4.99
		Narcobarbital	3.97
		Dinitrochlorobenzene; 1-Chloro-2,4-dinitrobenzene	3.91
		2'-(Benzyloxycarbonylaminomethyl)biphenyl-2-carboxylic acid	3.59
		2-(2-pyridyl)ethylamide	2.37
		Ethyl 4-nitrophenyl ethylphosphonate	
		3,4,5-trihydroxy-6-[(2-oxo-2H-chromen-8-yl)oxy]oxane-2-carboxylic	3.36
		acid	2.61
		Bromobenzene	
		4,4'-Biphenyldithiol	2.42
		Chalcolactone	2.42
			1.71

Table S2. Metabolites significantly increased (top 50) under heat stress treatment

Family	Number	Description	ratio
Amino acids, peptides, and analogues	2	Glucoraphenin	312.38
		Cefpimizole	44.72
Carbohydrates	3	Heparin	4565.54
		2'',3'',6''-Trigalloyliriflophenone 3-C-glucoside	43.64
		Zileuton O-glucuronide	43.62
Coumarins and derivatives	1	5,11-dihydroxy-6,8,19-trioxapentacyclononadeca-1(12),2(9),10,13(17)-tetraene-16,18-dione	40.86
Fatty Acyls	9	3-hydroxy-tetradecanedioic acid	59.11
		xi-5-Hydroxydodecanoic acid	55.95
		(3S)-citryl-CoA	55.43
		3-Hydroxynonyl acetate	51.48
		Ipurolic acid	48.75
		Cyclohex-1,5-diene-1-carboxyl-CoA	45.20
		13-hydroxy-tridecanoic acid	41.52
		3-hydroxy-tetradecanedioic acid	38.40
		Turpetholic acid B	34.48
Flavonoids	6	7-{4-[(6-carboxy-3,4,5-trihydroxyoxan-2-yl)oxy]-3,5-dimethoxyphenyl}-11-hydroxy-3-(4-hydroxyphenyl)-6-{[3,4,5-trihydroxy-6-(hydroxymethyl)oxan-2-yl]oxy}-2,8-dioxatricyclotrideca-1(13),2,4,6,9,11-hexaen-2-ylium	419.31
		Tricin 7-[feruloyl-(-2)-glucuronyl-(1-2)-glucuronide]	58.96
		3,4,5-trihydroxy-6-{[7-hydroxy-6-(3-methylbut-2-en-1-yl)-4-oxo-2-(2,4,5-trihydroxyphenyl)-3,4-dihydro-2H-1-benzopyran-5-yl]oxy}oxane-2-carboxylic acid	58.30
		(5-{8-[2-(3,4-dihydroxyphenyl)-3,7-dihydroxy-3,4-dihydro-2H-1-benzopyran-4-yl]-3,5,7-trihydroxy-3,4-dihydro-2H-1-benzopyran-2-yl}-2-hydroxyphenyl)oxidanesulfonic acid	47.90
		[2-({6-[(acetyloxy)methyl]-4,5-dihydroxy-2-{[5-hydroxy-2-(4-hydroxyphenyl)-4-oxo-4H-chromen-7-yl]oxy}oxan-3-yl}oxy)-4-hydroxy-4-(hydroxymethyl)oxolan-3-yl]oxidanesulfonic acid	42.91
		6-{[8-(2,4-dihydroxyphenyl)-2,2-dimethyl-6-oxo-2H,6H-pyrano[3,2-g]chromen-5-yl]oxy}-3,4,5-trihydroxyoxane-2-carboxylic acid	40.68
Nucleic acids and analogues	3	NADH	77.19
		UDP-D-apiose	57.34
		CMP-N-glycoloylneuraminate	50.69
Polyketides	7	[(6-{[5,7-dihydroxy-2-(4-oxocyclohexa-2,5-dien-1-ylidene)-2H-chromen-3-yl]oxy}-3,4-dihydroxy-5-{[3,4,5-trihydroxy-6-(hydroxymethyl)oxan-2-yl]oxy}oxan-2-yl)methyl][1-hydroxy-3-(4-hydroxy-3-methoxyphenyl)prop-2-en-1-ylidene]oxidanium	282.15
		Malvidin 3-O-(6-O-(4-O-caffeoyl-alpha-rhamnopyranosyl)-beta-glucopyranoside)	188.47
		Herbacetin 7-methyl ether 3-(2''-(E)-feruloylglucoside)	78.76
		Isohemsleyanoside	76.82
		Delphinidin 3-(2''-galloylgalactoside)	69.85
		Robinetinidol-(4alpha->8)-catechin-(6->4alpha)-robinetinidol	40.24
		Polystachin	40.06
Prenol lipids	1	Isopentenyl pyrophosphate	72.96
Purines	4	GDP-glucose	68.15

and derivatives			
		Xanthylic acid	52.18
		Adenosine diphosphate ribose	45.49
		ADP	36.04
Quinone	1	2-Hexaprenyl-6-methoxy-1,4-benzoquinone	64.89
Sphingolipids	1	C16 Sphingosine	88.90
Terpenoids	1	(1S,2S,4R,8R)-p-Menthane-1,2,8,9-tetrol	38.68
1,4-benzodiazepines	1	Quazepam	34.58
other	10	Coenzyme F420	169.26
		ADPribose 2'-phosphate	119.72
		Dephospho-CoA	65.77
		dTDP-D-glucuronate	61.60
		Quinolin-2,8-diol	55.46
		P1,P2-Bis(5'-adenosyl) triphosphate	45.38
		Watasenia luciferin	43.30
		3-Phosphinomethylmalate; 3-(Hydrohydroxyphosphorylmethyl)malate	35.88
		Protomycinolide IV	35.78
		11beta,17beta-Dihydroxy-9alpha-fluoro-17alpha-methyl-5beta-androstan-3-one	34.66

Table S3. OPLS-DA scatter plots of LC-MS data of *P. ostreatus* samples in each of the two groups

mode	group	R^2	Q^2	pvalue (R^2)	pvalue (Q^2)
pos	HS/No HS	0.9993	0.9646	0	0.005
pos	No HS+SA/No HS	0.9906	0.5836	0.03	0
neg	HS/No HS	0.9995	0.9733	0	0.01
neg	No HS+SA/No HS	0.9922	0.7837	0.005	0

$R^2 > 0.5$ and $Q^2 > 0.5$ indicated a good model.

Table S4. qRT-PCR primers used in this study

Target gene	primer
PLEOSDRAFT_1052690	F-RT-1052690：GGGGATTTGAAGAATAAGC
	R-RT-1052690：CAACCACATCAGCGAGTA
PLEOSDRAFT_168954	F-RT-168954：ACCTCTGTAGCAATCTCCC
	R-RT-168954：GTTTCGTGCCAAGTTCA
PLEOSDRAFT_1091186	F-RT-1091186：GGCAATCAACCACCAAG
	R-RT-1091186：TCGCTCAAGGAACCAGA
PLEOSDRAFT_1100329	F-RT-1100329：ACCGAAAGAGGACGATG
	R-RT-1100329: GCCAGTAAGTAGGTAAATAGG
PLEOSDRAFT_1052138	F-RT-1052138：CCGTGCTGGGTGTTATT
	R-RT-1052138：TCGGTTCGTCTCGTCAT
PLEOSDRAFT_1110516	F-RT-1110516：AACGGCACTAAGGATGA
	R-RT-1110516：CAAGAACCACAACACCAG
PLEOSDRAFT_1091553	F-RT-Mih1：ACCCGCCTCACATAACT
	R-RT-Mih1：CTGCCATCTTTCCCAAC
PLEOSDRAFT_1033113	F-RT-Rlm1：ACTGCTCAACCGACATCC
	R-RT-Rlm1：CTTCACCTTCCCATCCC
PLEOSDRAFT_1064366	F-RT- Swi4：CGCTGCTCCTAAACCTG
	R-RT- Swi4：GGCGTCATTGTTCCATC
PLEOSDRAFT_1113160	F-RT- Swi6：ACCACCGTCCACCTCCG
	R-RT- Swi6：ATCCGTGCGTCCAGTCC
PLEOSDRAFT_1054937	F-RT-1054937：CCGTCGTTGAAGATACCA
	R-RT-1054937：CGTTAGGCAAGCCGTTA
PLEOSDRAFT_1063335	F-RT-1063335：CTACTGCTGGCTACTTACCT
	R-RT-1063335：TGTTCCCTATTTCATTTCC
PLEOSDRAFT_1087442	F-RT-1087442：CGACCGATGTTTATGCTG
	R-RT-1087442：CGGAACTCAATGATGTAGAAG
PLEOSDRAFT_1065201	F-RT-1065201：ACCTTAATTCCTCATCTACCA
	R-RT-1065201：CCCATCTTTACCACTGTCC
PLEOSDRAFT_28823	F-RT-28823：GAGCATCTATCGCTTGTG
	R-RT-28823：CATGGACTCTGTCGTCTTA
PLEOSDRAFT_1058237	F-RT-1058237：TATCATTCTGTCGTTAGCC
	R-RT-1058237：TTTATTGTCTCCCTTGGT
PLEOSDRAFT_186160	F-RT-186160：TGAGTTTGGTGTAAGTTCG
	R-RT-186160：ATCGTCCCTGGGTATGG
PLEOSDRAFT_1093142	F-RT-1093142：GGGGCACTGAATAAGGC
	R-RT-1093142：TCCAGCGTAGTGATTGATA
PLEOSDRAFT_1060345	F-RT-1060345：ATTGGAAGGTCTTGCTC
	R-RT-1060345：TAACAGGTTGCGTGATG
actin	F-RT-ACT GTGCTGTTTTCCCTTCAATCG
actin	R-RT-ACT CTCCATATCGTCCCAGTTAGTG
